# Evaluation of the provision of nutrition in a South African provincial hospital

**DOI:** 10.1186/cc14474

**Published:** 2015-03-16

**Authors:** S Kudsk-Iversen, R Matos-Puig, R Naidoo, S Moorad

**Affiliations:** 1 Stanger Provincial Hospital, Stanger, South Africa

## Introduction

The provision of nutrition in the critical care unit (CCU) has shifted from nutrition support to nutrition therapy, and the potential benefits derived from this in the recovery of the critically ill is being explored [1]. We audited the management of nutrition in the CCU in a South African Hospital against the American Society of Parenteral and Enteral Nutrition Guidelines. Furthermore, we reviewed the knowledge and confidence of healthcare providers in the management of nutrition in the CCU.

## Methods

Retrospective data collection of patients admitted to a four-bed CCU over a 4-month period in 2013. A survey was distributed to different disciplines involved in patient nutrition in the CCU.

## Results

Seventy-two patients were admitted to the CCU during this time period, and notes were able for 44. Three paediatric patients were excluded. Twenty-nine patients stayed for 2 or more days (the audit population). The median age of the audit population was 38, 19 were female. Sixteen were postoperative admissions. The median APACHE II score of the patients with sufficient available data (*n *= 16) was 14 (range 6 to 34). The audit found that 21 of the patients had nutrition started in the CCU, with 15 having nutrition started within 48 hours. Only eight patients had a nutritional assessment done. A total of 45 responded to the survey: eight anaesthetists, 25 from surgical disciplines, seven CCU nurses, and five dieticians. All agreed that nutrition should be started in the first 48 hours, except from the surgeons only 14 (56%) agreed. The average self-rating of knowledge of nutrition management in the CCU (1 = lowest, 5 = highest) was 2.1 with the dieticians and CCU nurses showing the highest confidence with 3.4 and 2.6, respectively. The anaesthetists rated their knowledge at 1.9 and the surgeons rated themselves at 1.8.

## Conclusion

We found that there is poor management of nutrition in the CCU. This is paired with limited knowledge and low confidence in management amongst the attending staff. Evidence would suggest that the development and dissemination of clear hospital guidelines could improve rates of correct management [2]. However, the lack of uniform guidance based on strong evidence from the leading global authorities on nutrition suggests that, in order to improve implementation of adequate nutrition, more research is urgently required.
